# Supplementary material for: Netcombin: An algorithm for constructing optimal phylogenetic network from rooted triplets
Source: PLoS One. 2020 Sep 18;15(9):e0227842. doi: 10.1371/journal.pone.0227842 (PMC7500971; doi:10.1371/journal.pone.0227842)
Supplement: S4 File — (ZIP) [file pone.0227842.s004.zip › SequenceData-Triplet/sequenceData-10-20-40-Triplet2/data-(sample 5-length 500- num seq 40- m rate 10^-7)/4 seq.pdf]

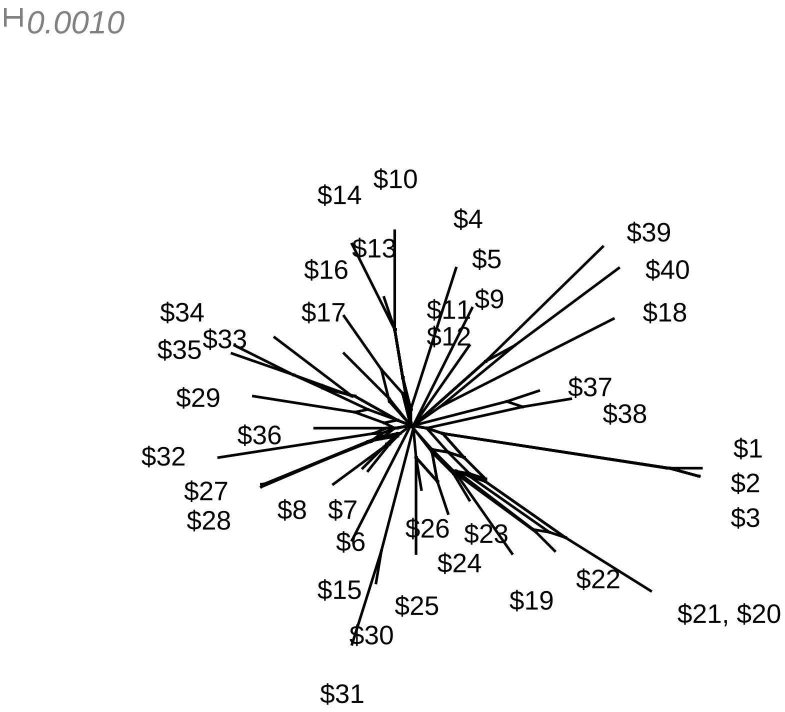
0.0010
$10
$14
$4
$39
$13
$5
$16
$40
$9
$11
$17
$18
$34
$12
$33
$35
$37
$29
$38
$36
$1
$32
$2
$27
$3
$8
$7
$28
$26
$23
$6
$24
$22
$15
$19
$25
$21, $20
$30
$31
